# Regulation of overexpression lncRNA ATP2B1-AS1 on lung adenocarcinoma progression

**DOI:** 10.1186/s13019-024-02507-2

**Published:** 2024-02-12

**Authors:** Shiyi Chen, Chao Huang, E Jin

**Affiliations:** Department of Medical Oncology Ward 1, The 4th People’s Hospital of Shenyang, No. 20, Huanghe South Street, Huanggu District, Liaoning, 110000 China

**Keywords:** lncRNA ATP2B1-AS1, Lung adenocarcinoma, miR-141-3p, Prognosis, Progression

## Abstract

**Background:**

LncRNA ATP2B1-AS1 (ATP2B1-AS1) is involved in the occurrence and development of various diseases, while the relationship between lung adenocarcinoma (LUAD) and ATP2B1-AS1 is unclear. This study was to investigate the expression of ATP2B1-AS1 in LUAD and its influence on survival and prognosis of patients.

**Methods:**

LUAD tissue samples from patients participating in this study were collected, and the expression levels of ATP2B1-AS1 and miR-141-3p in LUAD sampleswere detected by real-time quantitative polymerase chain reaction (RT-qPCR). The effect of ATP2B1-AS1 on the growth of A549 cells was investigated through cell counting kit-8 (CCK-8) and transwell experiments. Besides, the prognostic value of ATP2B1-AS1 in LUAD was assessed via Kaplan-Meier curve and multivariate Cox regression.

**Results:**

ATP2B1-AS1 was downregulated in LUAD tissues and cells, whereas miR-141-3p was upregulated. After pcDNA3.1-ATP2B1-AS1 was transfected into A549 cells, the proliferation ability of A549 cells was decreased, and the migration level and invasion of A549 cells were also inhibited. High expression of ATP2B1-AS1 sponge miR-141-3p exerted prognostic value.

**Conclusions:**

ATP2B1-AS1 sponge miR-141-3p alleviated the progression of LUAD, and ATP2B1-AS1 may be deemed as a prognostic marker for LUAD.

## Background

Lung adenocarcinoma (LUAD) is a common subtype of non-small cell carcinoma, which is a classification of lung cancer [1]. According to data from 2020, lung cancer ranks second in incidence and first in mortality [2]. In addition to surgical treatment, radiotherapy and drug-assisted therapy, targeted therapy and immunotherapy are also the main methods for the treatment of LUAD [3]. Although good clinical efficacy has been achieved, the clinical benefit population is still limited [4]. As far as the current medical level is concerned, the etiology of LUAD has not yet been definitively determined. A large number of medical data indicate that smoking, radiation, and the environment may become risk factors for LUAD [5]. However, the early clinical symptoms of the patients are not obvious, and more than 75% of the patients are prone to local infiltration or even distant metastasis in the middle and late stage, with poor prognosis and 5-year overall survival rate of less than 20% [6, 7]. Therefore, the feasibility of creative treatment and prognosis of LUAD needs to be explored.

LncRNA ATP2B1-AS1 (ATP2B1-AS1) is a 3,626 bp transhipment antisense RNA, located at CHR12:89,708,954 − 89,713,726 [8]. In recent decades, ATP2B1-AS1 has been reported to play regulatory and control functions in many diseases, suggesting that ATP2B1-AS1 may participate in disease progression [8, 9]. What’s more, a variety of LUAD-related lncRNAs have been confirmed to be involved in the treatment process and survival outcomes of patients, such as SATB2-AS1, BFSP2-AS1, LINC01089 and GAS6-AS1 [10–12]. Yao et al. used lncRNAs to establish 7-FIRLS signal and provided new hope for the prognosis of LUAD patients [13]. Wu et al. provided evidence that lncRNA CASC2 and miR-21/p53 axis mediate the proliferation and apoptosis of LUAD cells [14]. ATP2B1-AS1 has previously been linked to the development of colorectal cancer, but no studies on LUAD have been conducted [15]. Based on the effects of different expression levels of ATP2B1-AS1 on LUAD cells and progression, this study deepened the understanding of the pathological mechanism of LUAD.

The literature clarifies that lncRNAs sponge microRNAs (miRNAs), a class of endogenous RNAs that can promote or inhibit tumor growth [16]. For example, the LncRNA-SNHG14/NAP12/miRNA-3940-5p axis has been described as a diagnostic and prognostic marker for colorectal cancer, contributing to the management of colorectal development [17]. Targeting of the miRNA-30a-5p/KIF11 axis by VPS9D1-AS1 leads to the malignant progression of LUAD [18]. In this study, the expression and mechanism of ATP2B1-AS1 and its target miRNAs in LUAD were expounded to provide theoretical reference for ATP2B1-AS1 as a prognostic indicator for LUAD.

## Methods

### Included samples

The diseased tissue and normal tissue of 106 LUAD patients in The 4th People’s Hospital of Shenyang were collected as research samples. Inclusion criteria: patients were diagnosed as LUAD by pathologist; patients did not receive radiotherapy or chemotherapy before surgery; patients voluntarily agreed to participate in this study. Exclusion criteria: patients with other diseases or other types of lung cancer. The study was conducted under the guidance of the hospital Ethics Committee and with the consent of the patients. The included tissue samples were pathologist-confirmed as LUAD tissue or normal tissue, which was drained of blood immediately after obtaining the tissue, then frozen in liquid nitrogen and promptly kept at -80 °C.

### Culture and transfection of cells

LUAD cells including A549, H1792, NCI-H650 and NCI-H23 and control BEAS-2B cells were purchased from American Type Culture Collection (ATCC) as experimental strains for this study. DMEM medium (Dulbecco’s modified Eagle’s medium; Gibco, Shanghai) with fetal bovine serum (FBS) was configured for cell culture, and the incubator temperature was set at 37℃.

After the cells were cultured to logarithmic growth phase, A549 cells were transfected with empty vector or pcDNA3.1 or pcDNA3.1-ATP2B1-AS1 via Lipofectamine®3000 and divided into control group, pcDNA3.1 group and pcDNA3.1-ATP2B1-AS1 group (overexpression group). Similarly, A549 cells were collected 48 h after transfection in an incubator with a volume fraction of 5% CO_2_ at 37℃, and the transfection efficiency of the overexpression group was detected by PCR.

### Differential expression of ATP2B1-AS1 in LUAD

RNA was extracted from the sample tissue by grinding with TRIzol, and the RNA concentration and purity were determined spectrophotometrically. Complementary DNA (cDNA) was synthesized by reverse transcription using ReverTra Ace qPCR RT kit (Toyobo, Japan), and SYBR Green Realtime PCR Master Mix (Toyobo, Japan) was used to configure PCR reaction system for relative quantitative analysis.The reaction conditions were as follows: 95℃ for 1 min, 95℃ for 15 s, 60℃ for 30 s, with 40 cycles. Relative ATP2B1-AS1 levels were normalized to glyceraldehyde 3-phosphate dehydrogenase (GAPDH) and miR-141-3p levels were normalized to U6. The results were analyzed by 2^−ΔΔCT^ method for the expression of ATP2B1-AS1.

### CCK-8 analysis and transwell experiments

A549 cells of the transfected control group, pcDNA3.1 group and pcDNA3.1-ATP2B1-AS1 group were transferred to 96-well plates for culture, and CCK-8 solution was added at 0, 24,48,72 and 96 h after incubation. The absorbance value at 450 nm in each well was measured after incubation for 1 h.

A transwell chamber was placed in 24-well plate and Matrigel was added. After culturing for 4 h, DMEM medium was poured into the upper layer, and 1 × 10^4^ A549 cells were inoculated in this medium, while DMEM medium containing FBS was poured into the lower layer and saved in an incubator at 37 °C with a volume fraction of 5% CO_2_ for 24 h. After fixation with 4% paraformaldehyde, crystal violet staining was performed, and pictures were counted under a microscope to analyze the invasion level. The procedure for detecting the migration level of A549 cells was the same as above, but the addition of Matrigel was not required.

### Dual-luciferase reporter experiments

The existence of binding sites for ATP2B1-AS1 and miR-141-3p was found by prediction, and WT-ATP2B1-AS1 (wild-type) and MUT-ATP2B1-AS1 (mutant-type) were constructed. Similarly, WT-ATP2B1-AS1 or MUT-ATP2B1-AS1 were co-transfected into A549 cells with GenePharma-synthesized mimic NC, inhibitor NC, miR-141-3p mimic or miR-141-3p inhibitor, that supported by Lipofectamine® 3000, and luciferase activity was recorded.

### Statistical analysis

SPSS 23 and Graphpad 9 were used for data and visual analysis. The experimental data were expressed as mean ± standard deviation. The comparison between two groups of data was evaluated by Student’s t-test, while the comparison between multiple groups via one-way analysis of variance (ANOVA), and the correlation analysis by Spearman’s test. Kaplan-Meier survival curve and Cox regression were performed to analyze the prognosis and survival of LUAD patients. All experiments were carried out with biological repetition, and *P* < 0.05 was considered to be statistically significant.

## Results

### ATP2B1-AS1 was underexpressed in LUAD tissues and cells

**Fig. 1:**
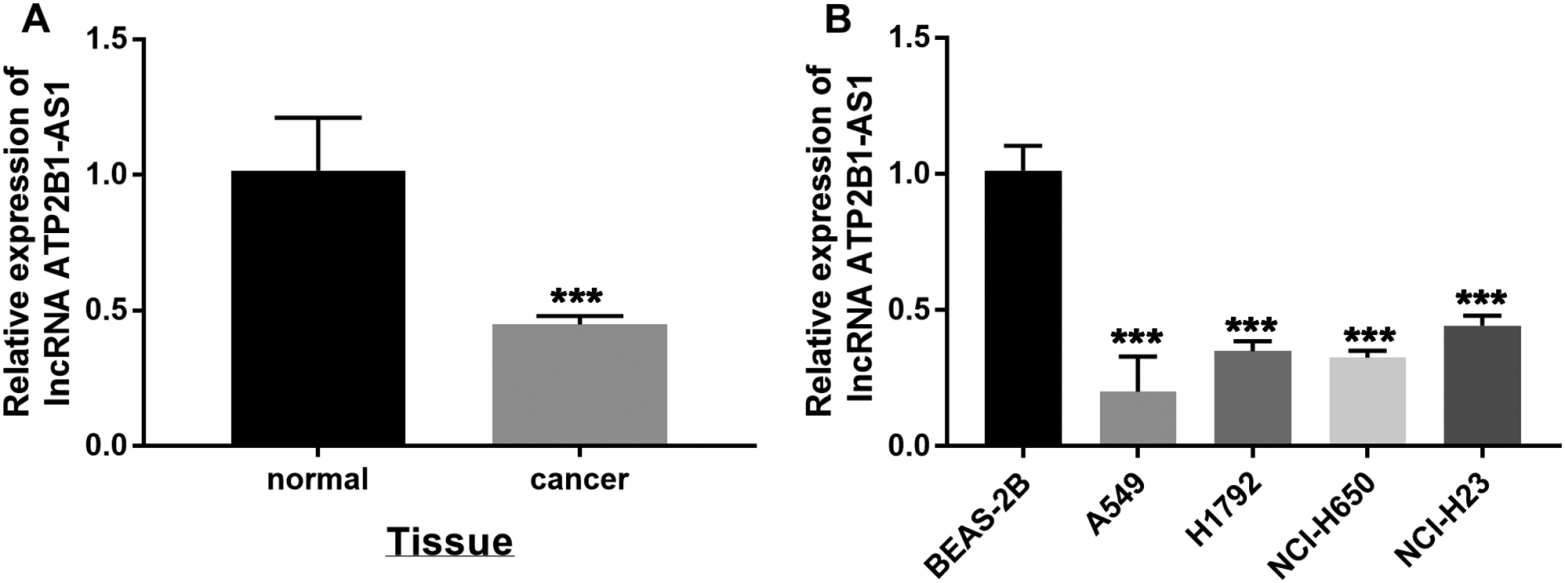
LncRNA ATP2B1-AS1 was down-regulated in LUAD. (**A**) Compared with normal tissues, ATP2B1-AS1 was decreased in LUAD tissues. (**B**) Low expression of ATP2B1-AS1 in A549, H1792, NCI-H650 and NCI-H23 cells. ***P < 0.001

To understand the effect of ATP2B1-AS1 on LUAD by measuring ATP2B1-AS1 levels in LUAD tissues and cells. The included tissue samples were tested, and ATP2B1-AS1 was decreased in LUAD tissues compared with normal tissues (Fig. 1A). ATP2B1-AS1 level was also higher in LUAD cells A549, H1792, NCI-H650, and NCI-H23 than in normal cells BEAS-2B (Fig. 1B).

### ATP2B1-AS1 slowed the malignant progression of LUAD

**Fig. 2:**
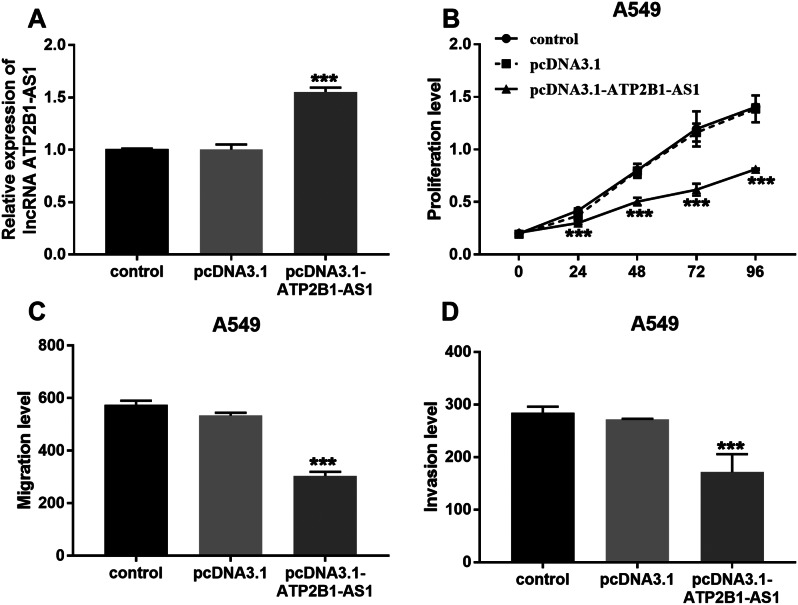
LncRNA ATP2B1-AS1 delayed the malignant progression of LUAD. (**A**) The level of ATP2B1-AS1 in A549 cells increased after transfection. (**B**) The CCK8 results of transfected A549 cells. (**C**) and (**D**) pcDNA3.1-ATP2B1 -AS1 attenuated the migratory and invasive abilities of A549 cells. ***P < 0.001

The pcDNA3.1-ATP2B1-AS1 was constructed to further explore the regulation of ATP2B1-AS1 on the growth of LUAD cells. The transfection results are shown in Fig. 2A, and the level of ATP2B1-AS1 in cells increased after the overexpression vector was constructed. Meanwhile, the overexpression group (pcDNA3.1-ATP2B1-AS1) attenuated the proliferation level of A549 cells and decreased their viability compared with the control group and the vector-transfected group (pcDNA3.1) in Fig. 2B. In addition, pcDNA3.1-ATP2B1-AS1 weaken the migratory performance and invasive abilities of A549 cells (Fig. 2C and 2D). That is, overexpression of ATP2B1-AS1 effectively inhibited the growth activity of LUAD cells.

### The mechanism of ATP2B1-AS1 sponged miR-141-3p

**Fig. 3:**
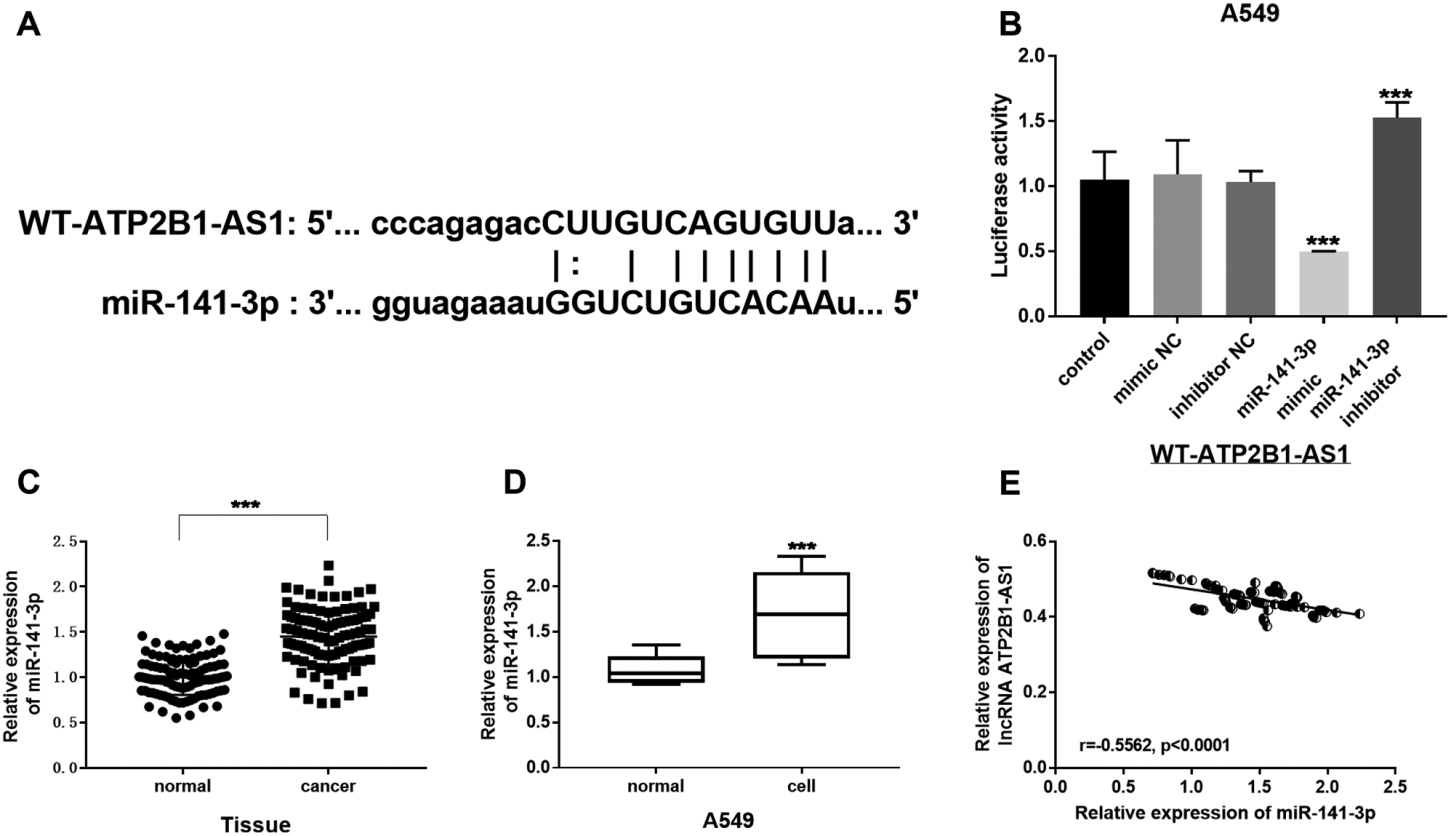
The mechanism of ATP2B1-AS1 sponged miR-141-3p. (**A**) ATP2B1-AS1 may interact with miR-141-3p. (**B**) Detection of luciferase activity in A549 cells. (**C**) and (**D**) The expression of miR-141-3p was increased in LUAD tissues and A549 cells. (**E**) ATP2B1-AS1 was negatively correlated with downstream miR-141-3p (*r* = -0.5562, *P* < 0.0001). ***P < 0.001

To explore the biological mechanism of ATP2B1-AS1 acting on downstream miR-141-3p to elucidate the regulation of LUAD. Figure 3A predicted the existence of binding sites between ATP2B1-AS1 and miR-141-3p. In Fig. 3B, the luciferase reporter gene suggested that WT-ATP2B1-AS1 reduced luciferase activity in A549 cells transfected with miR-141-3p mimic, while conversely, the luciferase activity of A549 cells with the miR-141-3p inhibitor increased. Through RT-qPCR detection, it was found that the miR-141-3p expression in LUAD tissues was increased, and the results were similar, miR-141-3p expression in A549 cells was higher than that in normal cells (Fig. 3C and D). Furthermore, Fig. 3E represented the negative correlation between ATP2B1-AS1 and downstream miR-141-3p (*r* = -0.5562, *P* < 0.0001). This proved that high expression of ATP2B1-AS1 targeting miR-141-3p slow disease progression in LUAD patients.

### The expression of ATP2B1-AS1 and the prognosis of patients

**Fig. 4:**
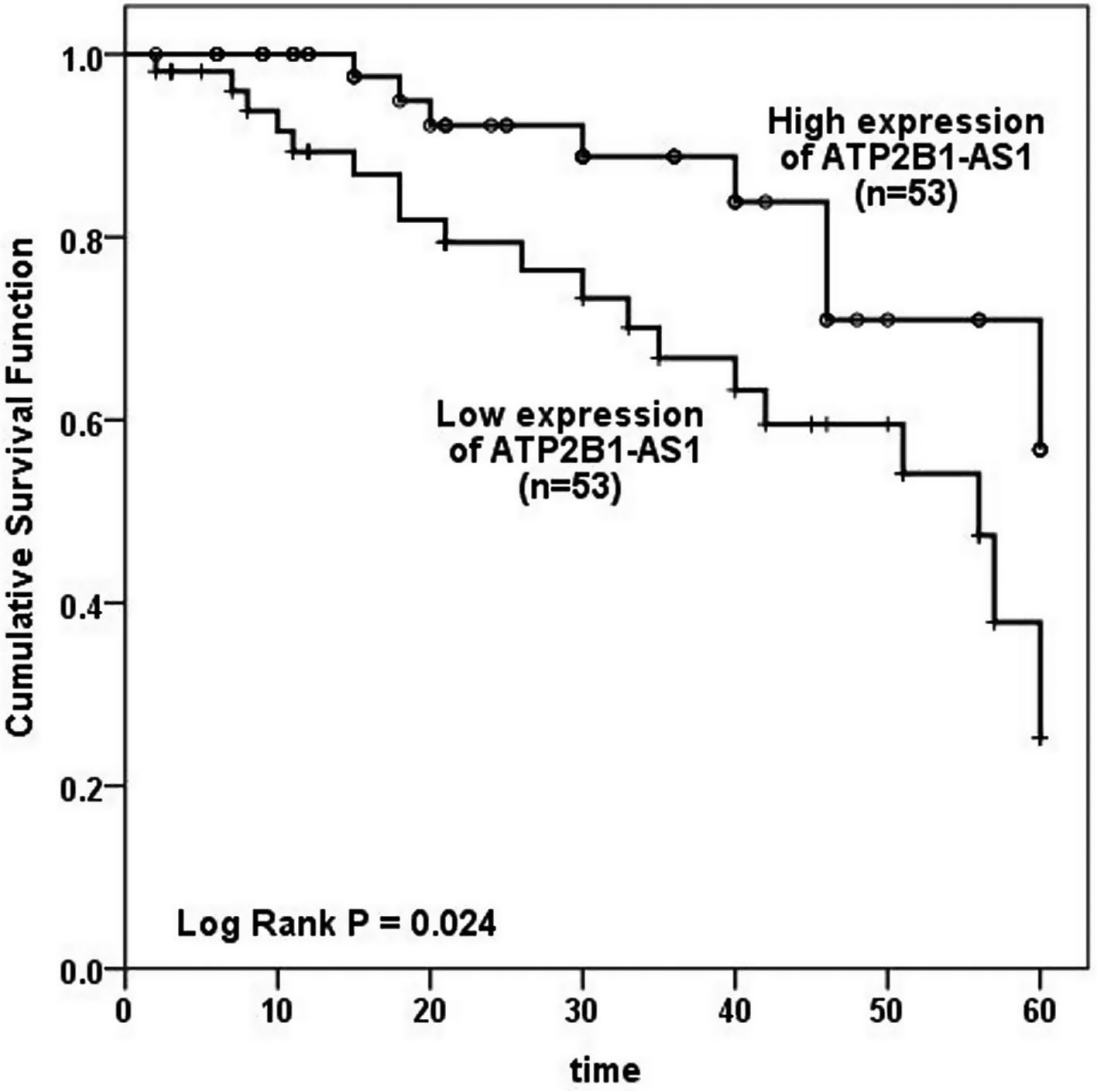
The expression of ATP2B1-AS1 predicted poor prognosis in LUAD patients. The Kaplan-Meier method suggested that the high expression of ATP2B1-AS1 was more conducive to the survival of patients (Log-Rank *P* = 0.024)

Referring to the Kaplan-Meier curve analysis, the survival rate of LUAD patients with low ATP2B1-AS1 expression was lower than that of the ATP2B1-AS1 high expression group (Log Rank *P* = 0.024; Fig. 4). The next multivariate Cox analysis revealed that ATP2B1-AS1 expression (95% CI = 2.136–20.120, *P* = 0.001), lymph node metastasis (95% CI = 1.055–11.205, *P* = 0.040) and TNM stage (95% CI = 1.056–5.624, *P* = 0.037) were independent prognostic factors of LUAD patients (Table 1).

**Table 1:** Multivariate Cox analyses of overall survival of prostate cancer patients

Characteristics	Multivariate Cox analysis		
	HR	95% CI	P
lncRNA ATP2B1-AS1	6.555	2.136–20.120	0.001
Age	1.154	0.522–2.547	0.724
Gender	1.318	0.557–3.120	0.530
Tumor size	1.128	0.505–2.522	0.769
Lymph node metastasis	3.439	1.055–11.205	0.040
Differentiation	1.380	0.507–3.758	0.529
TNM stage	2.437	1.056–5.624	0.037

## Discussion

Lung cancer, as a common cancer, has high morbidity and mortality, and different subtypes of lung cancer have different clinical characteristics and prognoses. LUAD is prone to local invasion, distant metastasis and drug resistance, resulting in unsatisfactory therapeutic effects [19]. Statistics indicated that more than 1 million people die each year from LUAD, which is the leading cause of death worldwide [20]. Nowadays, with the innovation and breakthroughs of bioinformatics, molecular biology, immunology and other technologies, promising methods such as molecular targeting and immunosuppression have been used in clinical practice, but the mortality rate of LUAD patients remains high [21, 22]. Therefore, it is also a new challenge to explore the specific prognostic markers of LUAD.

ATP2B1-AS1, also known as intergenic long non-protein-coding RNA 936 (LINC00936), is underexpressed in various diseases. Ren and colleagues assessed patients with diabetic retinopathy and learned that down-regulation of ATP2B1-AS1 expression could ameliorate cell permeability by targeting miR-4729, thereby alleviating retinopathy in patients [9]. Shu et al. stated that LINC00936 was downregulated in ovarian cancer tissues and that the combination of miR-221-3p with high expression of LINC00936 controlled the occurrence of ovarian cancer [23]. Similar results, ATP2B1-AS1 expression was decreased in LUAD tissues and cells. After constructing and overexpressing ATP2B1-AS1 in A549 cells, it was found that the proliferative capacity, migration performance, and invasion level of LUAD cells were inhibited. It is referenced that in LUAD-related literature, HCG11 expression was also reduced and the growth of LUAD cells was inhibited by IGF2BP2/LATS1 [24]. What’s more, highly expressed lncRNA ARAP1-AS1 promoted the progression of LUAD by mediating miR-8068/CEACAM5 [25].Subsequently, downstream target genes of ATP2B1-AS1 were predicted to show that there were binding sites between ATP2B1-AS1 and miR-141-3p, which might regulate LUAD through sponge miR-141-3p. Kang et al. confirmed that miR-141-3p was significantly elevated in Th17 cells and participated in differentiation, providing evidence for the pathogenesis of autoimmune diseases [26]. Yang and colleagues revealed the upregulation mechanism of miR-141-3p in endometrial cancer and predicted the key role of miR-141-3poverexpression in carcinogenesis and prognosis [27]. In addition, miR-141-3p was confirmed to be differentially expressed as well in human diseases such as polycystic ovary syndrome [28], clear cell renal cell carcinoma [29], and colorectal cancer [30]. The above studies demonstrated the ability of ATP2B1-AS1 to affect the development of different types of tumors and the prognosis of patients through multiple mechanisms. The results of this study revealed that miR-141-3p was higher in LUAD tissues and cells than in the matched control group by PCR detection. The luciferase activity assay implied that ATP2B1-AS1 targets miR-141-3p to participate in the development of LUAD, and ATP2B1-AS1 negatively regulates miR-141-3p. Kaplan-Meier and multivariate analysis showed that high expression of ATP2B1-AS1 improved the survival time of patients. In dong’s study of breast cancer in vivo and in vitro, low expression of MEG3 sponge miR-141-3p affected cell proliferation performance and apoptosis, which was negatively correlated with high expression of miR-141-3p [31]. Meanwhile, circKEAP1 inhibited the progression of LUAD by targeting miR-141-3p and opened a new idea for the treatment of LUAD [32], which was consistent with our study. The previous analysis confirmed that ATP2B1-AS1 specifically binds to miR-141-3p to participate in the development of LUAD and play a regulatory role in the prognosis of patients from the aspects of cell function and biological mechanism.

Obviously, the deficiency lies in that the downstream target of miR-141-3p has not been further studied, and the samples need to be expanded for follow-up exploration, so as to more clearly understand the regulatory pathway and prognostic value of ATP2B1- AS1 on LUAD. In addition, we also need to design animal assays in subsequent studies to grasp more clinical information and increase the reliability of research results.

## Conclusions

In conclusion, ATP2B1-AS1 was markedly underexpressed in LUAD and elucidated poor prognosis of LUAD, which may serve as a valuable prognostic biomarker. In addition, overexpression of ATP2B1-AS1 suppressed the proliferation capacity, migration level and invasion of A549 cells.

## Data Availability

The datasets used and/or analysed during the current study are available from the corresponding author on reasonable request.

## Abbreviations

ATCC: American Type Culture Collection
ATP2B1-AS1: LncRNA ATP2B1-AS1
CCK-8: cell counting kit-8
LINC00936: long non-protein-coding RNA 936
LUAD: lung adenocarcinoma
miRNAs: microRNAs
RT-qPCR: real-time quantitative polymerase chain reaction
